# Metabolic engineering of oleaginous yeast *Yarrowia lipolytica* for limonene overproduction

**DOI:** 10.1186/s13068-016-0626-7

**Published:** 2016-10-11

**Authors:** Xuan Cao, Yu-Bei Lv, Jun Chen, Tadayuki Imanaka, Liu-Jing Wei, Qiang Hua

**Affiliations:** 1 State Key Laboratory of Bioreactor Engineering, East China University of Science and Technology, 130 Meilong Road, Shanghai, 200237 People’s Republic of China; 2 Shanghai Collaborative Innovation Center for Biomanufacturing Technology, 130 Meilong Road, Shanghai, 200237 People’s Republic of China

**Keywords:** Limonene, *Yarrowia lipolytica*, Neryl diphosphate synthase 1, Limonene synthase

## Abstract

**Background:**

Limonene, a monocyclic monoterpene, is known for its using as an important precursor of many flavoring, pharmaceutical, and biodiesel products. Currently, d-limonene has been produced via fractionation from essential oils or as a byproduct of orange juice production, however, considering the increasing need for limonene and a certain amount of pesticides may exist in the limonene obtained from the citrus industry, some other methods should be explored to produce limonene.

**Results:**

To construct the limonene synthetic pathway in *Yarrowia lipolytica*, two genes encoding neryl diphosphate synthase 1 (NDPS1) and limonene synthase (LS) were codon-optimized and heterologously expressed in *Y. lipolytica*. Furthermore, to maximize limonene production, several genes involved in the MVA pathway were overexpressed, either in different copies of the same gene or in combination. Finally with the optimized pyruvic acid and dodecane concentration in flask culture, a maximum limonene titer and content of 23.56 mg/L and 1.36 mg/g DCW were achieved in the final engineered strain Po1f-LN-051, showing approximately 226-fold increase compared with the initial yield 0.006 mg/g DCW.

**Conclusions:**

This is the first report on limonene biosynthesis in oleaginous yeast *Y. lipolytica* by heterologous expression of codon-optimized *tLS* and *tNDPS1* genes. To our knowledge, the limonene production 23.56 mg/L, is the highest limonene production level reported in yeast. In short, we demonstrate that *Y. lipolytica* provides a compelling platform for the overproduction of limonene derivatives, and even other monoterpenes.

**Electronic supplementary material:**

The online version of this article (doi:10.1186/s13068-016-0626-7) contains supplementary material, which is available to authorized users.

## Background

Natural compound monoterpenes are C10 compounds that consist of two isoprene units. Monoterpenes belong to a large family of plant secondary metabolites with valuable applications including using as biofuels, feedstocks for pharmaceutical and other industrial product syntheses, and flavors and fragrances [4, 13, 23]. Limonene, a monocyclic monoterpene, is famous for its citrus-like olfactory properties. Limonene is also an important precursor of many flavor and medicinal compounds such as perillyl alcohol (POH), carvone, and menthol [1, 13]. Limonene has three isomers: d-limonene, dl-limonene, and l-limonene. They all consist in natural plants, while d-limonene is the most widespread [32]. D-limonene is considered as GRAS (generally recognized as safe) material by the US Food and Drug Administration [19]. Furthermore, d-limonene is widely used as a flavoring or fragrance agent in pharmaceuticals, foods, and beverages [27]. Currently, d-limonene has been produced via fractionation from essential oils or as a byproduct of orange juice production, but considering the increasing need for limonene as a polymer and as a jet fuel, the citrus industry may not be able to meet future demands. In the meantime, a certain amount of pesticides may exist in the limonene obtained from the citrus industry, so it is necessary that some other methods should be explored to produce limonene [10, 13].

**Fig. 1:**
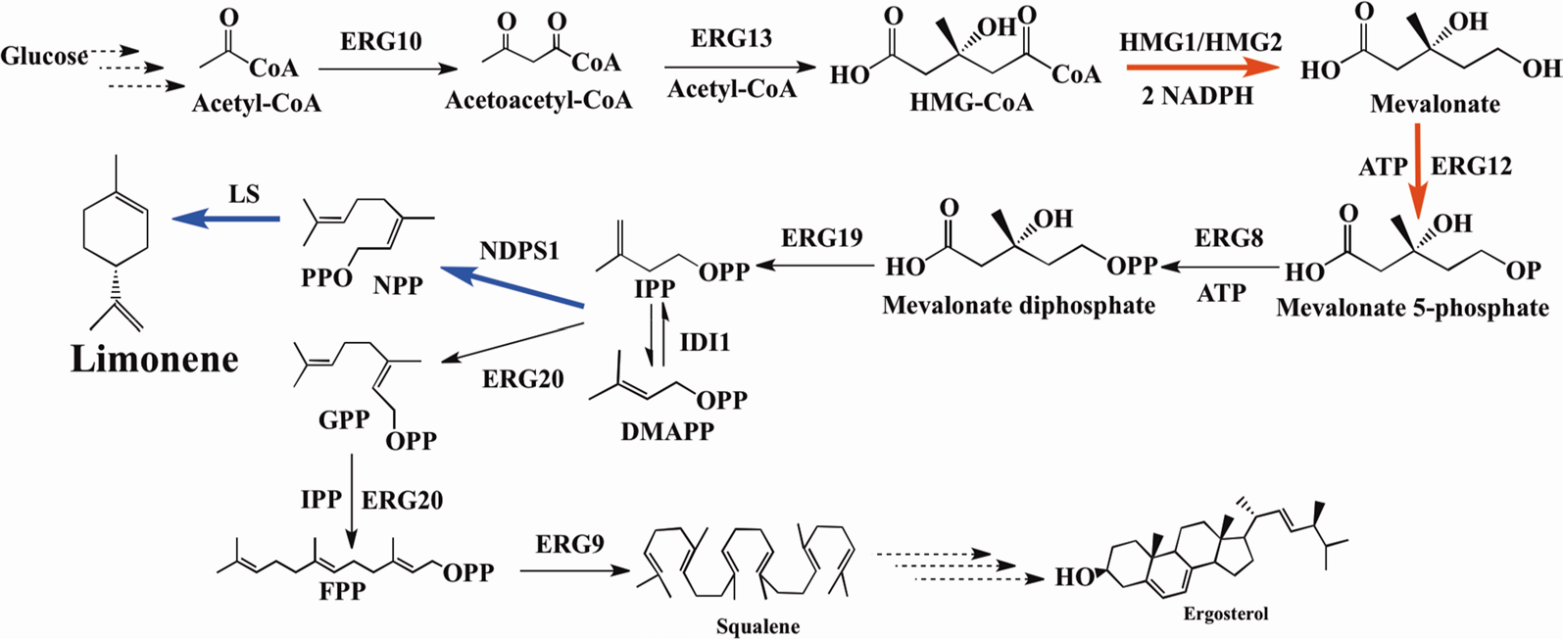
Biosynthesis pathway for limonene production in *Y. lipolytica.* IPP and DMAPP are converted to NPP by neryl diphosphate synthase 1 (NDPS1) and then NPP is further converted to limonene by limonene synthase (LS). *Blue arrows* represent that the pathways were exogenously integrated in *Y. lipolytica*, while *red arrows* represent that the pathways were overexpressed in *Y. lipolytica*.* Single arrows* represent the one-step conversions, while* triple arrows* represent multiple steps

Metabolic engineering of microorganisms to produce natural products seems to be a good choice. Many terpenoids have been produced by various kinds of microorganisms, such as lycopene produced in *Yarrowia lipolytica*; miltiradiene and sclareol produced in *Saccharomyces cerevisiae*; dammarenediol-II produced in *Pichia pastoris*; β-carotene produced in *Escherichia coli* and so on [6, 17, 21, 28, 35]. Limonene biosynthesis has been widely studied in other microorganisms including *E. coli* [1, 11, 33] and *S. cerevisiae* [4, 13]. In yeast, isopentenyl diphosphate (IPP) and its isomer dimethylallyl diphosphate (DMAPP), the two common building blocks, are the precursors of all terpenoids and they are derived from the mevalonic acid (MVA) pathway, and geranyl diphosphate (GPP) is the direct precursor of monoterpenes catalyzed by geranyl/farnesyl diphosphate synthase (ERG20) [7] (Fig. 1). Limonene can be produced by introducing one key enzyme, limonene synthase (LS) into the pathway based on GPP [4, 13]. LS catalyzes the intramolecular cyclization of GPP to gain limonene.

*Yarrowia lipolytica* as a non-conventional yeast is considered non-pathogenic and generally regarded as safe (GRAS). The availability of suitable metabolic engineering tools and the full genome sequence of *Y. lipolytica* make it a favorable host for producing pharmaceutical and food additives, and some studies have verified natural products produced in this strain [3, 21, 34]. *Yarrowia lipolytica* has the ability to grow to high biomass yield on simple substrates. A wide range of compounds can be produced by *Y. lipolytica* from glycerol, a side product of biodiesel production [8, 21, 25]. In this study, we aim to construct a limonene biosynthesis pathway in *Y. lipolytica* and then metabolically engineer this strain for efficient production of limonene. To reach the target, two genes encoding neryl diphosphate synthase 1 (*NDPS1*) and limonene synthase (*LS*) were codon-optimized and heterologously expressed in *Y. lipolytica*. Secondly, to enhance limonene production, several genes involved in the MVA pathway were overexpressed, either in different copies of the same gene or in combination. Lastly, the optimized addition of pyruvic acid and dodecane further improved limonene production. By combining metabolic engineering and optimization of genes expression, an approximately 226-fold increase in limonene productivity was obtained in comparison with the initial strain.

## Methods

### Strains, vectors, chemicals, and culture media

The vectors and strains used in this study are listed in Tables 1 and 2, respectively. The auxotrophic *Y. lipolytica* strain Po1f was used as the target gene expression host in this study. This *Y. lipolytica* strain and the plasmids pINA1269 and pINA1312 have been previously described by Madzak et al. [18]. *Yarrowia lipolytica* cells were cultured at 30 °C in YPD medium (1 % yeast extract, 2 % peptone, and 2 % glucose) or YNB medium (0.67 % yeast nitrogen base without amino acids, 1 % glucose, and 1.6 % agar). The rich medium YPD was used for strain activation and fermentation, while the synthetic medium YNB, lacking leucine and uracil where appropriate, was used for the screening of transformants. *Escherichia coli* strain JM109 or DH5α was used as a host for plasmid proliferation and construction of recombinant vectors. The cells were grown in Luria–Bertani (LB) complete medium (0.5 % yeast extract, 1 % tryptone, and 1 % NaCl) at 37 °C for 12 h. Suitable antibiotics or nutrients were added when necessary at the following final concentrations: ampicillin, 100 mg/L; kanamycin 50 mg/L; leucine 0.3 g/L. Plasmid extraction, gel extraction, and purification of DNA were conducted using assay kits from Sangon Biotech Co., Ltd. (Shanghai, China). Primer synthesis was conducted by GeneRay Biotech Co., Ltd. (Shanghai, China). Pyruvic acid and dodecane were purchased from TCI Development Co., Ltd. (Shanghai, China).

**Table 1:** Vectors used in this study

Vectors	Description	Source
pINA1312	*Y. lipolytica*-integrative plasmid, hp4d promoter, XPR2 terminator, ura3d1 selection marker, KmR	[22]
pINA1269	*Y. lipolytica*-integrative plasmid, hp4d promoter, XPR2 terminator, LEU2 selection marker, AmpR	[22]
p1312-tLS	Constitutively expressed truncated *LS* gene	This study
p1312-tNDPS1	Constitutively expressed truncated *NDPS1* gene	This study
p1312wLN	Constitutively expressed *tLS* and *tNDPS1* genes	This study
p1312LN	Constitutively expressed codon-optimized *tLS* and codon-optimized *tNDPS1* genes	This study
p1269tHMG1	Constitutively expressed *tHMG1* gene	This study
p1269HMG1	Constitutively expressed *HMG1* gene	This study
p1269IDI1	Constitutively expressed *IDI1* gene	This study
p1269ERG8	Constitutively expressed *ERG8* gene	This study
p1269ERG10	Constitutively expressed *ERG10* gene	This study
p1269ERG12	Constitutively expressed *ERG12* gene	This study
p1269ERG19	Constitutively expressed *ERG19* gene	This study
p1269tLS	Constitutively expressed *tLS* gene	This study
p1269tNPDS1	Constitutively expressed *tNDPS1* gene	This study
p1269tHMG1tHMG1	Constitutively expressed *tHMG1* gene of two copies	This study
p1269tHMG1tHMG1tHMG1	Constitutively expressed *tHMG1* gene of three copies	This study
p1269HMG1HMG1	Constitutively expressed *HMG1* gene of two copies	This study
p1269IDI1IDI1	Constitutively expressed *IDI1* gene of two copies	This study
p1269IDI1IDI1IDI1	Constitutively expressed *IDI1* gene of three copies	This study
p1269HMG1IDI1	Constitutively expressed *HMG1* and *IDI1* genes	This study
p1269HMG1ERG8	Constitutively expressed *HMG1* and *ERG8* genes	This study
p1269HMG1ERG10	Constitutively expressed *HMG1* and *ERG10* genes	This study
p1269HMG1ERG12	Constitutively expressed *HMG1* and *ERG12* genes	This study
p1269HMG1ERG19	Constitutively expressed *HMG1* and *ERG19* genes	This study
p1269HMG1tLS	Constitutively expressed *HMG1* and *tLS* genes	This study
p1269HMG1tNPDS1	Constitutively expressed *HMG1* and *tNDPS1* genes	This study

**Table 2:** Strains used in this study

Strains	Description	Source
*Escherichia coli*		
DH5α, JM109	For construction of recombinant vectors	Invitrogen
*Yarrowia lipolytica*		
Po1f	leu2^−^, ura3^−^	[2]
Po1f-LN-000	Po1f cells harboring p1312LN	This study
Po1f-LN-001	Po1f cells harboring p1312LN and p1269tHMG1	This study
Po1f-LN-002	Po1f cells harboring p1312LN and p1269tHMG1tHMG1	This study
Po1f-LN-003	Po1f cells harboring p1312LN and p1269tHMG1tHMG1tHMG1	This study
Po1f-LN-004	Po1f cells harboring p1312LN and p1269HMG1	This study
Po1f-LN-005	Po1f cells harboring p1312LN and p1269HMG1HMG1	This study
Po1f-LN-006	Po1f cells harboring p1312LN and p1269IDI1	This study
Po1f-LN-007	Po1f cells harboring p1312LN and p1269IDI1IDI1	This study
Po1f-LN-008	Po1f cells harboring p1312LN and p1269IDI1IDI1IDI1	This study
Po1f-LN-011	Po1f cells harboring p1312LN and p1269HMG1tLS	This study
Po1f-LN-021	Po1f cells harboring p1312LN and p1269HMG1tNDPS1	This study
Po1f-LN-031	Po1f cells harboring p1312LN and p1269HMG1ERG8	This study
Po1f-LN-041	Po1f cells harboring p1312LN and p1269HMG1ERG10	This study
Po1f-LN-051	Po1f cells harboring p1312LN and p1269HMG1ERG12	This study
Po1f-LN-061	Po1f cells harboring p1312LN and p1269HMG1ERG19	This study
Po1f-LN-071	Po1f cells harboring p1312LN and p1269HMG1IDI1	This study

### Plasmids construction

The gene encoding d-limonene synthase (*LS*, GenBank ID: AY055214.1) from *Agastache rugosa* and gene encoding neryl diphosphate synthase 1 (*NDPS1*, GenBank ID: NM_001247704.1) from *Solanum lycopersicum* were codon-optimized and synthesized by GeneRay Biotech. The transit peptides of both genes were removed according to the literatures [5, 26]. The truncated genes were named *tLS* and *tNDPS1*, respectively. The *tLS* gene and *tNDPS1* gene were cloned into p1312 with primers P1/P2 and P3/P4 to obtain vectors p1312-tLS and p1312-tNDPS1, respectively (Table 3). Then the expression cassette P-tLS-T was cloned into p1312-tNDPS1 with primers P5/P6 to obtain vector p1312LN. The genes *HMG1*, *IDI1*, *ERG8*, *ERG10*, *ERG12*, *ERG19* amplified from the genome of Po1f with *tLS* and *tNDPS1* were cloned into p1269 with primers P7/P8, P9/P10, P11/P12, P13/P14, P15/P16, P17/P18, P1/P2, and P3/P4 to obtain the respective vectors p1269HMG1, p1269IDI1, p1269ERG8, p1269ERG10, p1269ERG12, p1269ERG19, p1269tLS, p1269tNPDS1. Afterwards, the expression cassettes P-tHMG1-T, P-HMG1-T, P-IDI1-T, P-ERG8-T, P-ERG10-T, P-ERG12-T, P-ERG19-T, P-tLS-T, and P-tNDPS1-T were cloned into p1269tHMG1, p1269HMG1, p1269IDI1 with primers P19/P20 to obtain p1269tHMG1tHMG1, p1269HMG1HMG1, p1269IDI1IDI1, p1269HMG1tLS and p1269HMG1tNPDS1, p1269HMG1ERG8, p1269HMG1ERG10, p1269HMG1ERG12, p1269HMG1ERG19, p1269HMG1IDI1, respectively. Then the expression cassettes P-tHMG1-T and P-IDI1-T were cloned into p1269tHMG1tHMG1 and p1269IDI1IDI1 with primers P21/P22 to obtain p1269tHMG1tHMG1tHMG1 and p1269IDI1IDI1IDI1, respectively. All the plasmids were constructed using the One Step Cloning Kit from Vazyme Biotech Co., Ltd. (Nanjing, China).

**Table 3:** Primers used in this study

Primer	Primer sequence (5′–3′)
P1	ACAACCACACACATCCACGTGATGCGACGATCCGGTAACTACTCCCCTT (*Pml*I)
P2	TTAGTTTCGGGTTCCCACGTGCTAGGCGAAAGGCTGGAACAGGCAA (*Pml*I)
P3	ACAACCACACACATCCACGTGATGTCCGCCCGAGGTCTCAACAAAA (*Pml*I)
P4	TTAGTTTCGGGTTCCCACGTGCTAGTAGGTGTGGCCACCGAATCGT (*Pml*I)
P5	AGATAGAGTCGACAAAGGCCTGCTAGCTTATCGATACGCGTGCATG (*Stu*I)
P6	TGTACACCGAGAAACAGGCCTCATCTCACTTGCGTATGTATGGAAA (*Stu*I)
P7	ACAACCACACACATCCACGTGATGCTACAAGCAGCTATTGG (*Pml*I)
P8	TTAGTTTCGGGTTCCCACGTGCTATGACCGTATGCAAATATT (*Pml*I)
P9	ACAACCACACACATCCACGTGATGACGACGTCTTACAGCGA (*Pml*I)
P10	TTAGTTTCGGGTTCCCACGTGCTACTTGATCCACCGCCGAA (*Pml*I)
P11	ACAACCACACACATCCACGTGATGACCACCTATTCGGCTCC (*Pml*I)
P12	TTAGTTTCGGGTTCCCACGTGCTACTTGAACCCCTTCTCGA (*Pml*I)
P13	ACAACCACACACATCCACGTGATGCGACTCACTCTGCCCCG (*Pml*I)
P14	TTAGTTTCGGGTTCCCACGTGCTACTCGACAGAAGAGACCT (*Pml*I)
P15	ACAACCACACACATCCACGTGATGGACTACATCATTTCGGC (*Pml*I)
P16	TTAGTTTCGGGTTCCCACGTGCTAATGGGTCCAGGGACCGA (*Pml*I)
P17	ACAACCACACACATCCACGTGATGATCCACCAGGCCTCCAC (*Pml*I)
P18	TTAGTTTCGGGTTCCCACGTGCTACTTGCTGTTCTTCAGAG (*Pml*I)
P19	CGAGGCAGCAGATCCACTAGTAGCACCGCCGCCGCAAGGAATGG (*Spe*I)
P20	GCGGCCGCATAGGCCACTAGTCTGTCAAACATGAGAATTCGG (SpeI)
P21	CTCTCAAGGGCATCGGTCGACAGCACCGCCGCCGCAAGGAATGG (*Sal*I)
P22	CGCATAAGGGAGAGCGTCGACCTGTCAAACATGAGAATTCGG (*Sal*I)
P23	CGGCATCCGCTTACAGAC
P24	GGAGGCATCAGTGACCAAA
P25	CATTAGGAAGCAGCCCAGTA
P26	GAGATCGTCAAGGGTTTG
P27	CATAAGTGCGGCGACGAT
P28	CTACTACTGGGCTGCTTCCTA

Restriction sites are underlined

### Strain construction

p1312LN was linearized with *Not*I and then was transformed into competent Po1f cells using the kit, Frozen-EZ yeast transformation II. After transformation, cells were cultured on YNB medium plates with leucine added. The right colonies verified with primers P1/P2 and P3/P4 were named Po1f-LN-000. Then, p1269tHMG1, p1269tHMG1tHMG1, and p1269tHMG1tHMG1tHMG1 were linearized with ApaI while p1269HMG1, p1269HMG1HMG1, p1269IDI1, p1269IDI1IDI1, and p1269IDI1IDI1IDI1 were linearized with *Bsr*GI. All these linearized plasmids were transformed into competent Po1f-LN-000 cells, respectively. These cells were cultured on YNB medium plates and the right colonies, verified with primers P23/P24, P25/P26, and P27/P28 were named Po1f-LN-001, Po1f-LN-002, Po1f-LN-003, Po1f-LN-004, Po1f-LN-005, Po1f-LN-006, Po1f-LN-007, and Po1f-LN-008, respectively. Afterwards, p1269HMG1tLS, p1269HMG1tNPDS1, p1269HMG1ERG8, p1269HMG1ERG10, p1269HMG1ERG12, p1269HMG1ERG19, and p1269HMG1IDI1 were linearized separately with BsrGI and transformed into Po1f-LN-000. After verification, the right colonies obtained were therefore named Po1f-LN-011, Po1f-LN-021, Po1f-LN-031, Po1f-LN-041, Po1f-LN-051, Po1f-LN-061, and Po1f-LN-071, respectively.

### Yeast cultivation

YPD medium was used to cultivate the engineered strains. All strains were firstly inoculated into 10 mL culture tubes containing 2 mL medium, and grown at 30 °C at 220 rpm to reach an OD_600_ of about 1.0. The cultures were started by inoculating 50 mL medium containing 1 mL dodecane as organic extractant phase with the preculture [30] and the initial OD_600_ was 0.01. Strains were cultured at 30 °C and 220 rpm for 3 days. All the flask fermentation results represented the mean ± S.D. of three independent experiments. At the same time, to investigate the influence of the addition of pyruvic acid in vitro, pyruvic acid of different concentrations 2, 4, and 8 g/L were added to the medium of strain Po1f-LN-051, respectively. Furthermore, effects of the amount of dodecane on limonene synthesis by strain Po1f-LN-051 were also surveyed, with dodecane varying from 2 to 10 % (volume/volume) and constant pyruvic acid concentration of 4 g/L.

### Analysis

Optical densities at 600 nm (OD_600_) were measured using a Shimadzu UV-1800 spectrophotometer (Shimadzu Co., Kyoto, Japan). 5 mL of wet cell culture was harvested, washed twice with distilled water, and centrifuged at 12,000 rpm for 5 min. The upper layer was discarded and dry cell weight was determined by measuring the weight of cell pellets which were dried at 105 °C for 48 h.

For limonene detection, the dodecane layers were collected and myrcene was used as an internal standard. The dodecane overlay samples were mixed with myrcene by 4:1 (v/v). Then the mixture (1 μL) was analyzed by GC/MS using an Agilent System 6890 gas chromatograph coupled to an Agilent 5975 quadrupole mass selective detector (EI) (Agilent Technologies, Santa Clara, CA), equipped with a HP-5 (30 m × 0.25 mm, 0.25 µm film thickness) GC column. The GC oven temperature program was as follows: 100 °C for 1 min, a ramp of 0.5 °C/min to 102 °C, and then a rise to 280 °C in 5 min. The split ratio was 20:1. Limonene and myrcene standards (purchased from Sigma-Aldrich) were used for quantification.

For squalene extraction, 1.8 mL of fermentation broth was collected and resuspended with 600 μL of 20 % KOH/50 % ethanol solution then voltexed at once using a multi holder for 5 min at the fastest rate. After that, the tubes were placed into boiling water for 5 min then cooled in ice water. HPLC grade hexane 600 μL was added to the tubes, which was then voltexed using a multi holder for 5 min. The two phases were separated by centrifugation at 12,000 rpm for 5 min at 4 °C. After centrifugation, 400 μL of top hexane layer was transferred into the new 1.5 mL eppendorf (EP) tubes and evaporated using a vacuum dryer for 15 min at a low dry rate, after that 50 μL of HPLC grade ethanol and 450 μL of HPLC grade acetonitrile were added to the tubes. Squalene was quantified with the Shimadzu LC-20A (Shimadzu Co., Kyoto, Japan) equipped with C18 column (Phenomenex Kinetex 5 μm C18) and a UV detector. The wavelength was 195 nm and mobile phase was 100 % acetonitrile with 2 mL/min flow rate and a column temperature of 35 °C. Squalene standard (purchased from TCI Biotech) was used for quantification.

#### Inhibitory effects of d-limonene on *Y. lipolytica* Po1f

A fixed volume of serially diluted d-limonene in ethanol ranging from 100 to 3000 mg/L was added to YPD medium with 0.5 % Tween 80 v/v. Tween 80 was dissolved into the medium to increase the solubility of d-limonene. YPD medium contained 0.5 % Tween 80v/v and ethanol was regarded as control.

## Results and discussion

### Construction of the limonene synthetic pathway in *Y. lipolytica*

Though *Y. lipolytica* possesses a native MVA pathway which can supply the intermediates DMAPP and IPP (Fig. 1), it cannot produce limonene because of the absence of limonene synthase. Consequently, the gene encoding d-limonene synthase (*LS*) was first amplified from the cDNAs of *Agastache rugosa*. Meanwhile, to overcome the possible expression problem a codon-optimized artificial *LS* gene was also designed and amplified. The integrative vector pINA1312 carrying either the wild-type *LS* gene or the codon-optimized artificial *LS* gene was successfully integrated into the chromosome of Po1f strain, respectively. Unfortunately, after three-day cultivation of these two engineered strains, no limonene could be detected in the dodecane layers by GC–MS (data not shown), based on the relative retention time and total ion mass spectral comparison with the external standard.

**Fig. 2:**
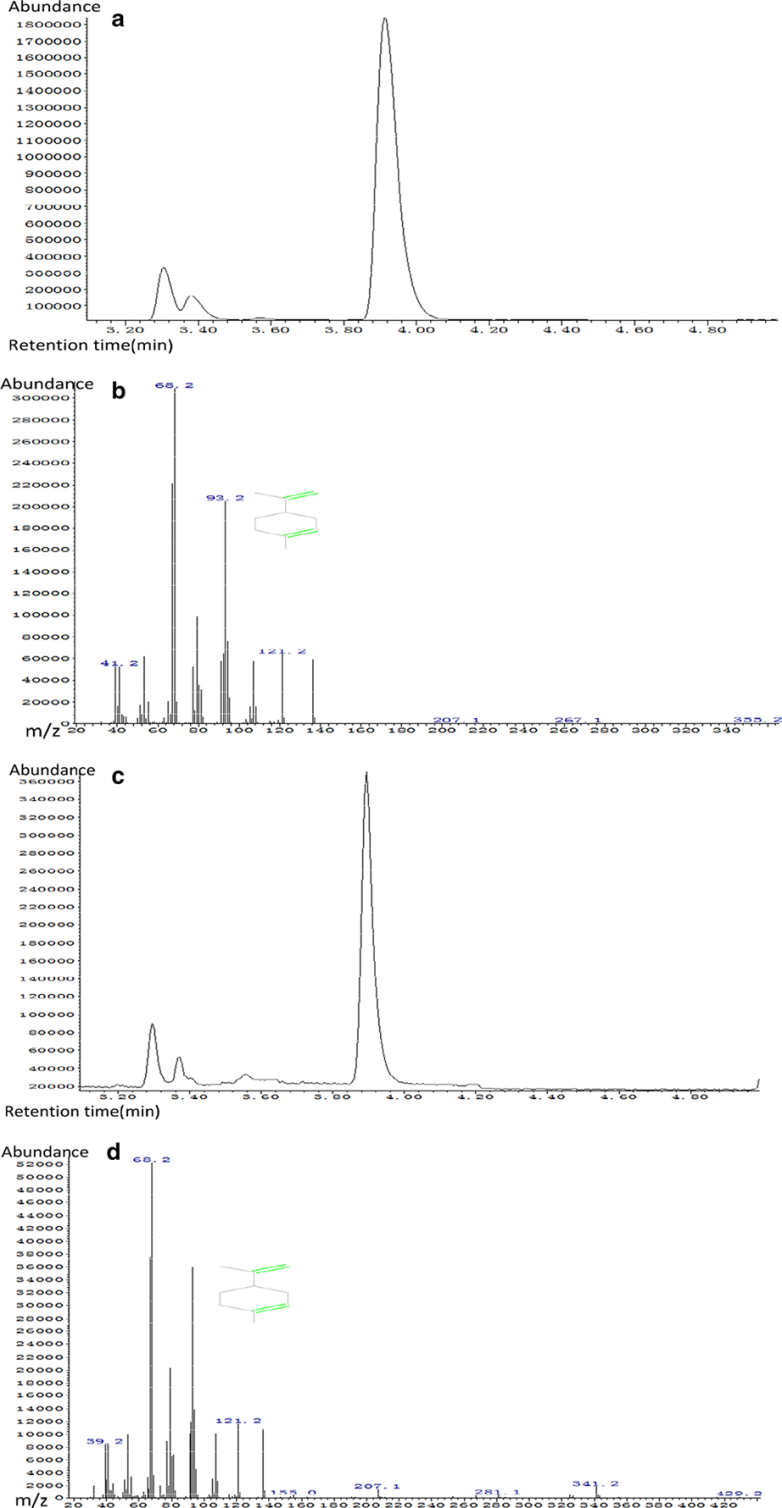
GC–MS analysis of limonene from the dodecane phase of the cultures in engineered *Y. lipolytica*. The strain was cultivated in YPD medium for 72 h. **a** Limonene standard; **b** Mass spectrum of limonene standard; **c** Limonene obtained in YPD medium containing 4 g/L pyruvic acid of Po1f-LN-051; **d** Mass spectrum of limonene obtained in YPD medium containing 4 g/L pyruvic acid of Po1f-LN-051

**Fig. 3:**
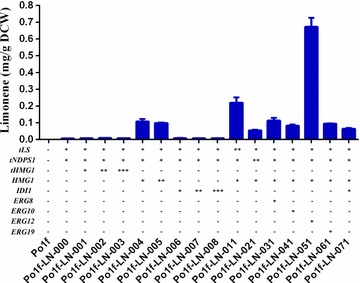
Quantitative analysis of limonene production in engineered *Y. lipolytica*. Limonene productions in the engineered strains Po1f-LN-000, Po1f-LN-001, Po1f-LN-002, Po1f-LN-003, Po1f-LN-004, Po1f-LN-005, Po1f-LN-006, Po1f-LN-007, Po1f-LN-008, Po1f-LN-011, Po1f-LN-021, Po1f-LN-031, Po1f-LN-041, Po1f-LN-051, Po1f-LN-061, Po1f-LN-071. All strains were cultured in YPD medium for 3 days. Three repeats were performed for each strain, and *error bars* represent standard deviations

Geranyl diphosphate (GPP) has been generally regarded as the substrate for monoterpenes, while very little was reported about NPP, the isomer of GPP. Recently, monoterpenes in the glandular trichomes of tomato were found to be synthesized from NPP precursor rather than GPP [26]. Another study showed that NPP instead of GPP was the substrate of limonene in *S. cerevisiae* [16]. Therefore, we hypothesized that NPP might also be the major substrate for limonene biosynthesis in *Y. lipolytica*. To address the precursor problem, codon-optimized *NDPS1* gene, which encodes NDPS1 catalyzing the conversion of DMAPP and IPP to NPP in *Solanum lycopersicum*, were integrated into the chromosome of *Y. lipolytica* Po1f together with the codon-optimized *LS* gene to obtain strain Po1f-LN-000. Dodecane layers were collected on the third day and tested by GC/MS. Limonene (*m/z* 68.1, 93.1, and 136.5) was monitored at 3.9 min (Fig. 2). The engineered strain Po1f-LN-000 produced limonene in detectable quantities at 0.006 mg/g DCW and was therefore used as the initial limonene-producing strain in this study (Fig. 3). Thus, using codon-optimized gene *tLS* from *Agastache rugosa* and *tNDPS1* from *S. lycopersicum*, the biosynthetic pathway for limonene production was successfully constructed in *Y. lipolytica*. The results also demonstrated that introduction of NPP pathway is essential for limonene production and the genes derived from plants were necessary to be codon-optimized when expressed in *Y. lipolytica*. After this initial success in metabolic engineering of *Y. lipolytica* for limonene production, further stepwise improvements were attempted using this Po1f-LN-000 strain. The optimized nucleotide sequences of truncated *LS* gene and truncated *NDPS1* gene were provided in Additional file 1.

### Improving limonene production by overexpressing HMG-CoA reductase gene

The enzyme 3-hydroxy-3-methylglutaryl-coenzyme A (HMG-CoA) reductase is well known as the major rate-limiting enzyme of the mevalonate (MVA) pathway in many organisms, including yeasts. Overexpression of the catalytic domain of the HMGR (producing a form of the enzyme that lacks the membrane-binding region, namely truncated HMG-CoA reductase gene or *tHMG1*) in yeasts has been shown to boost production of isoprenoid biosynthesis [9, 24, 29]. Furthermore, in the oleaginous yeast *Y. lipolytica*, it was also reported that the integration of *HMG1* into the engineered strain led to a 6.9-fold increase in lycopene content after 96 h of cultivation, probably attributable to the increased supply of precursors [21]. Thus, it was determined whether overexpression of *HMG1* could increase limonene production in limonene-producing *Y. lipolytica* cells. For this purpose, heterologous *tHMG1* gene from *S. cerevisiae* and homologous *HMG1* gene from *Y. lipolytica* were overexpressed under the control of hy4d promoter in the limonene-producing strain Po1f-LN-000, as showed in Fig. 3, resulting in the recombinant strains Po1f-LN-001 and Po1f-LN-004, respectively. Limonene production was only improved around 30 % in the heterologous recombinant strain Po1f-LN-001, while homologous recombination of *HMG1* significantly enhanced the limonene-producing level. In the fermentation broths of the strain Po1f-LN-004, the limonene titer (or content) increased from 0.006 mg/g DCW to 0.11 ± 0.01 mg/g DCW, 18-fold higher than the control strain Po1f-LN-000 (Fig. 3). These results did confirm that the production of limonene was accumulated by the duplication of HMG-CoA reductase gene and, more importantly, the overexpression of homologous *HMG1* was more effective to increase the biosynthesis of limonene in *Y. lipolytica*.

Further enhancing the *HMG1* copy number might lead to further improvement of the limonene production. To validate the hypothesis, the plasmids carrying extra gene copies were introduced into Po1f-LN-000, yielding another type of recombinant strains (Po1f-LN-005) that contain *HMG1* of two copies. We found that the two copies of *HMG1* did not necessarily improve limonene production, instead a slight decrease in limonene titer was observed. Similar phenomenon also happened for strains Po1f-LN-002 harboring two copies of *tHMG1* and Po1f-LN-003 with three copies of *tHMG1* (Fig. 3). These results implied that it was not necessary to increase the copy number of HMG-CoA reductase gene, and single copy of homologous *HMG1* was good enough for limonene overproduction in *Y. lipolytica*.

### Effects of re-engineering of limonene synthesis on limonene production

Since there was only a single copy of *tLS* and *tNDPS1* gene in chromosome with relatively low expression strength, limonene synthase and neryl diphosphate synthase enzymes might be rate limiting for limonene production. In order to investigate the effects of increased activities of these enzymes on limonene production, plasmids p1269HMG1tLS and p1269HMG1tNDPS were transformed into strain Po1f-LN-000 (Po1f cells harboring p1312LN and p1269HMG1) to generate strain Po1f-LN-011 with two copies of *tLS* and strain Po1f-LN-021 with two copies of *tNDPS1*, respectively. Shake flask cultures of strain Po1f-LN-004 and the engineered strains of Po1f-LN-011and Po1f-LN-021 were carried out and the limonene productions were compared (Fig. 3). In contrast to the slight decrease in limonene production in strain Po1f-LN-021, the final limonene concentration by Po1f-LN-011 doubled in comparison with that of the engineered strain Po1f-LN-004, suggesting that strengthening the conversion of precursor NPP to limonene plays a more important role in improving the production. In contrast to the effectiveness of enhancing *tLS* expression, strain with extra copy of *tNDPS1* produced less amount of limonene than that of Po1f-LN-004 strain, indicating that single expression of *tNDPS1* was more efficient in converting IPP and DMAPP into NPP.

### Effects of overexpression of gene encoding isopentenyl diphosphate isomerase on limonene production

The gene *IDI1* (Fig. 1) encodes for an isopentenyl diphosphate isomerase that catalyzes an essential step in the sterol pathway, the isomerization of IPP to DMAPP. A number of works focused on the overexpression of *IDI* gene to improve isoprenoid accumulation in *E. coli* [14, 20, 31]. In *S. cerevisiae*, an extra copy of *IDI1* was introduced into the genome leading to a 50 % increase in geraniol yield. Furthermore, overexpression of *IDI1* using a multi-copy plasmid in the engineered strains increased geraniol yield 1.45-fold [15]. In another study, overexpression of *IDI1* increased the monoterpene cineole production by twofold, and approximately fivefold increase was obtained when *IDI1* was expressed from a high copy plasmid in the engineered *S. cerevisiae* strains [12]. In order to investigate the effects of overexpressing homologous *IDI* on limonene production, the plasmids carrying different copies of *IDI* were transformed into Po1f-LN-000 strain resulting in strains Po1f-LN-006 (single copy), Po1f-LN-007 (two copies), and Po1f-LN-008 (three copies), respectively. The data showed that single copy of *IDI1* gene could only increase the limonene concentration by 30 %. However, there was no obvious difference in limonene production with the increase in copy number of the *IDI1* gene. Although one extract *IDI1* integration could enhance the limonene biosynthesis in *Y. lipolytica*, the improvement was limited compared with the overexpression of *HMG1* (Fig. 3). When both *HMG1* and *IDI1* genes were overexpressed in Po1f-LN-000 (resulting in strain Po1f-LN-071), interestingly, we observed a slight decrease in limonene production instead of the production enhancement in comparison with Po1f-LN-004, suggesting that *IDI1* might exert limited influence on limonene accumulation in *Y. lipolytica*.

### Optimization of mevalonate pathway improved limonene production

**Fig. 4:**
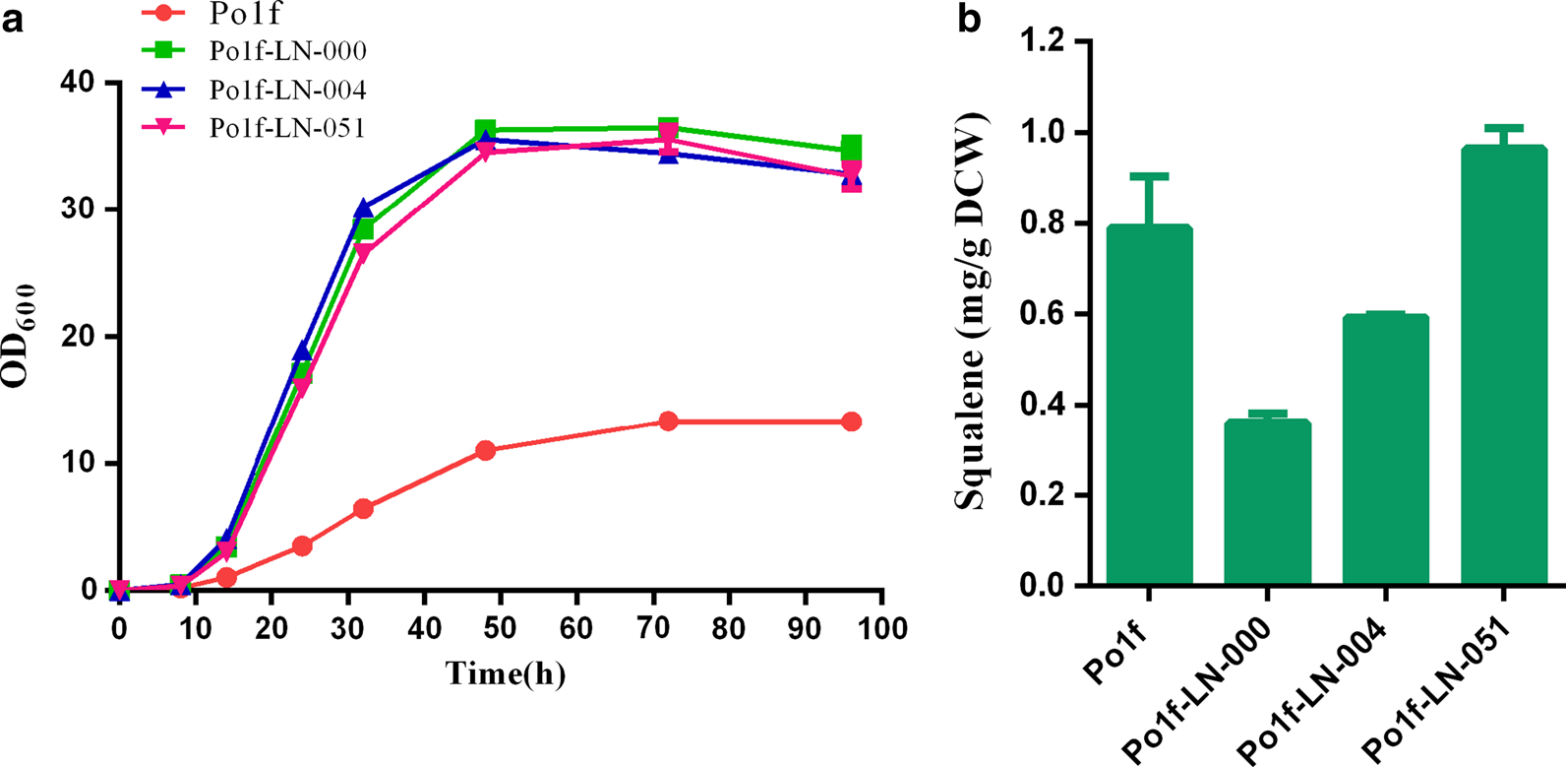
**a** The OD600 values of strains Po1f, Po1f-LN-000, Po1f-LN-004, and Po1f-LN-051 cultured in YPD medium, measured at 0, 8, 14, 24, 32, 48, 72, and 96 h. **b** Squalene production in strains Po1f, Po1f-LN-000, Po1f-LN-004, Po1f-LN-051 cultured in YPD medium for 5 days

Our previous work has already shown that the homologous overexpression of *HMG1* was efficient in boosting limonene production in *Y. lipolytica*. In addition to the overexpression of the *HMG1* gene alone, we also emphasized on the co-overexpression of the *HMG1* gene together with other genes involved in the MVA pathway (e.g., *ERG8* encoding phosphomevalonate kinase, *ERG10* encoding acetoacetyl-CoA thiolase, *ERG12* encoding mevalonate kinase, and *ERG19* encoding mevalonate diphosphate decarboxylase), in order to further explore potential and efficient strategies for improving limonene productivity. The plasmids p1269HMG1ERG8, p1269HMG1ERG10, p1269HMG1ERG12, and p1269HMG1ERG19 were integrated into Po1f-LN-000 separately, resulting strains Po1f-LN-031, Po1f-LN-041, Po1f-LN-051, and Po1f-LN-061, respectively. As shown in Fig. 3, among all of the above recombinant strains, the improved limonene production could only be observed for the strain Po1f-LN-051 in which *ERG12* and *HMG1* were co-overexpressed. One hundred and 12- and 6-fold increases in limonene content were obtained in Po1f-LN-051 in comparison with those obtained by the control strains of Po1f-LN-000 and Po1f-LN-004, respectively. The results suggested the significant importance of mevalonate biosynthesis and its subsequent phosphorylation in supplying an enhanced carbon flux for the improved production of MVA pathway-related metabolites. It has not yet been reported that the overexpression of the *ERG12* gene is favorable to the synthesis of monoterpene. Actually, when both the *HMG1* and *ERG12* genes were overexpressed, we also observed a 2.5-fold increase in the content of squalene, a shunt biosynthetic product (i.e., 0.36 mg/g DCW and 0.96 mg/g DCW in strains Po1f-LN-000 and Po1f-LN-051, respectively), while the growth rates of these strains did not vary too much (Fig. 4).

### Effects of pyruvic acid and dodecane addition

**Fig. 5:**
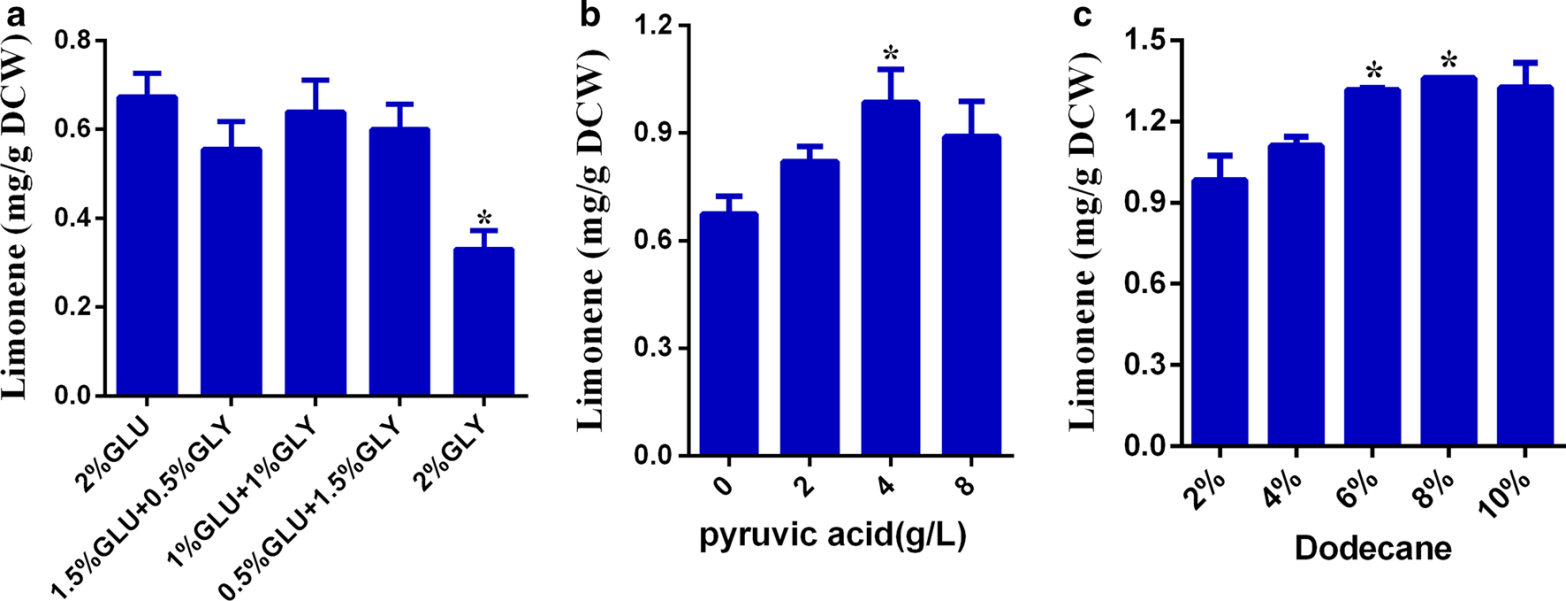
Effects of different combinations of glucose and glycerol as substrate and heterologous addition of pyruvic acid and dodecane on the production of limonene. **a** Glucose of 2, 1.5, 1, 0.5, 0 % were respectively combined with glycerol of 0, 0.5, 1, 1.5, 2 % as substrate in the YP medium of strain Po1f-LN-051. **b** Pyruvic acid was added to the YPD medium of strain Po1f-LN-051 to a final concentration of 0, 2 , 4, or 8 g/L. **c** Dodecane was added to the YPD medium of strain Po1f-LN-051 with 4 g/L pyruvic acid as the auxiliary carbon source and the proportion of dodecane was from 2 to 10 %. Three repeats were performed for each medium, and *error bars* represent standard deviations. *t* tests were conducted to evaluate statistical significance at *p* < 0.05. Particularly, for (**a**) the* asterisk* shows statistical significance between 2 % glucose and other combinations of glucose and glycerol, for (**b**) the *asterisk* shows statistical significance between YPD medium containing 0 g/L pyruvic acid and other concentrations of pyruvic acid, and for (**c**) the *asterisk* shows statistical significance between YPD medium containing 2 % dodecane and other proportions of dodecane

Based on the successes in genetic modification of *Y. lipolytica* for improving limonene biosynthesis, extensive efforts have been made in the optimization of culture medium to further increase limonene productivity. Among several key influential factors, effects of pyruvic acid and dodecane were investigated in this study. Pyruvic acid is a substrate of the DXP pathway and previous studies indicated that the addition of pyruvic acid as an auxiliary carbon source could increase amorphadiene and limonene accumulation in *E. coli* [10, 36]. In order to evaluate the effect of the amount of pyruvic acid on the growth of *Y. lipolytica* and limonene production, shake flask cultures with initial pyruvic acid concentrations varying from 0 to 8 g/L in YPD medium were conducted and compared. Although no obvious differences were observed for cell growth, the limonene contents were increased and 4 g/L of initial pyruvic acid seemed to favor the limonene production best (Fig. 5b). We also tested the use of glycerol as a sole carbon source or auxiliary carbon source. However, limonene productions were decreased apparently only as a sole carbon source in comparison with other combinations (Fig. 5a). The reason may be that the promoter when glycerol was used as substrate resulted in the lower expression of genes and reduced the production of limonene.

Limonene is produced and secreted by *Y. lipolytica* into the medium. Usually dodecane is added into the culture medium to enrich limonene. To investigate the amount of dodecane on limonene production, the cultures were performed with different initial dodecane concentrations varying from 2 to 10 % in YPD medium with 4 g/L pyruvic acid as the auxiliary carbon source. Maximum production of limonene in recombinant cells was observed when dodecane concentration was 8 %, and no significant differences on cell growth were found in all cases. With the optimized pyruvic acid and dodecane concentrations, a limonene production of 23.56 mg/L and 1.36 mg/g DCW was achieved, approximately 226-fold higher than those of the control strain Po1f-LN-000 (with only codon-optimized *LS* and *NDPS1* genes overexpressed) (Fig. 5c). These results demonstrated that both the addition of pyruvic acid and the appropriate dodecane concentration were important in enhancing limonene production by *Y. lipolytica*.

#### Inhibitory effects of d-limonene on *Y. lipolytica*

**Fig. 6:**
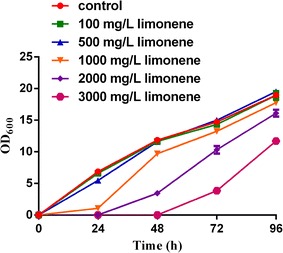
Inhibitory effects of d-limonene on *Y. lipolytica*. A fixed volume of serially diluted d-limonene in ethanol ranging from 100 to 3000 mg/L was added to YPD medium which contained 0.5 % Tween 80 (v v-1), YPD medium contained 0.5 % Tween 80 (v v-1) and ethanol was regarded as control. Po1f was used as the trial strain

As seen from the Fig. 6, yeast growth was similar to the control when the concentration of limonene in the medium was lower than 500 mg/L. The cell growth was significantly inhibited when limonene concentration was higher than 500 mg/L.

Limonene biosynthesis has been widely studied in *E. coli* and *S. cerevisiae*. In *E. coli*, the highest limonene production was 435 mg/L from simple sugars using a heterologous mevalonate pathway. Furthermore, the purification of limonene produced in *E. coli* was also explored recently [1, 4, 13, 33]. In *S. cerevisiae*, limonene trapped in a dodecane phase resulted in the recovery of 0.028 mg/L (+)-limonene and 0.060 mg/L (−)-limonene in strains expressing a mutant *ERG20* enzyme and the truncated *Citrus* and *Perilla* synthases, respectively. Another study showed that the best limonene titer obtained in *S. cerevisiae* was 1.48 ± 0.22 mg/L when a truncated *HMG1* and *upc2*-*1* were expressed with codon-optimized citrus (+)-limonene synthase in supplemented YP medium. Although the yield of limonene obtained in our study is lower than that in *E. coli*, considering the non-GRAS status of *E. coli*, it is of great commercial interest for GRAS status organism *Y. lipolytica* to produce limonene. In addition, the limonene production of the engineered strain in this study is the highest reported to date in yeasts, which might prompt further research on the biosynthesis of various terpenoids in *Y. lipolytica*.

## Conclusion

Limonene, an important monoterpene, is used as a precursor of many flavoring, pharmaceutical, and biodiesel products and majorly supplied limitedly in plant source. Recently, microbial production of valuable chemicals by economically efficient bioprocesses has emerged as an attractive alternative way. Here, our work presented the first report of the limonene biosynthetic pathway in oleaginous yeast *Y. lipolytica* by heterologous expression of codon-optimized *tLS* and *tNDPS1* genes. Specifically, we optimized the MVA pathway and re-engineered the limonene biosynthetic pathway for further stepwise improvement of limonene production capacity. Combining this metabolic engineering strategy with the optimization of medium in flask culture, a maximum limonene titer and content of 23.56 mg/L and 1.36 mg/g DCW (the highest reported in yeasts) was achieved in this study, showing approximately 226-fold increase compared with the initial yield of 0.006 mg/g DCW. As a conclusion, we demonstrated that *Y. lipolytica* could be a compelling platform for a feasible, scalable, and economic route to the overproduction of limonene derivatives, and even other monoterpenes. In our next experiments, fermentation strategies will be developed based on the engineered strains to obtain enhanced production. We will be focusing on several issues, such as optimizing the medium compositions and fermentation parameters, selecting suitable solvent and antifoam.

## Additional file

10.1186/s13068-016-0626-7 The optimized nucleotide sequences of truncated *LS* gene and truncated *NDPS1* gene.

## Abbreviations

DMAPP: dimethylallyl pyrophosphate
IPP: isopentenyl pyrophosphate
GPP: geranyl diphosphate
NPP: neryl diphosphate
MVA: mevalonate
NDPS1: neryl diphosphate synthase
LS: limonene synthase
