# Supplementary material for: Metabolic engineering of oleaginous yeast Yarrowia lipolytica for limonene overproduction
Source: Biotechnol Biofuels. 2016 Oct 11;9:214. doi: 10.1186/s13068-016-0626-7 (PMC5057495; doi:10.1186/s13068-016-0626-7)
Supplement: Supplementary file 1 — 10.1186/s13068-016-0626-7 The optimized nucleotide sequences of truncated LS gene and truncated NDPS1 gene. [file 13068_2016_626_MOESM1_ESM.doc]

**Metabolic Engineering of Oleaginous Yeast *Yarrowia lipolytica* for**

**Limonene Overproduction**

**Additional file 1**

**Nucleotide sequences of truncated LS and NDPS1 gene**

Note that these nucleotide sequences were codon-optimized based on the codon bias of Y. lipolytica, and the initiating ATG codon and the stop codon of the reading frame are in capital letters.

Truncated *LS* gene

ATGgccctgaagatgacctccgtcgtcatgcagatggccatccccaccaagctcgccaacttcgtgcacaactctgacaacaacaagtctctcaagctggtgcgaaacgtgtccaccacctgcacctccgccgctaccccccgactgcgactgcccgtgtgctgctcctcttcctcctcttcttcctctcagctgcctaccattgagcgacgatccggtaactactccccttctcgatgggacgtggacttcatccagtctctcaactctgactaccaggaagagcgacacacccgacgagcctctgaactgatcacccaggtgaagatgctcatggagaaagaaaccaccgaccctattcgacagctggagctgattgacgacctacaacgactgggactgtctgaccacttccagaacgagttcaaagagattctcaacaccatctatctggacaacaagtactacaacattaacattatgcgagaagaatcccgagacctgtactctaccgccctcgctttccgactgctccgagagcacggtttccaggtcgcccaagaagtgttcgagtgcttcaagaacgaagagggtgacttcaaggcttccctgattgacgacacccgaggtctcctacaactgtacgaggcctctttcctgttcaaggaaggtgagaacaccctggagatcgcccgagagttcaccaccaagatcctccaagagaagctgaagggtgacgagattgacgacaacctcctgtcttctattcgatactccctggagattcctaactactggtccgtcgtgcgacccaacgtgtctgtgtggattgacgagtaccgaaagcgatctgacatgaaccccgtcgtgctagaattagccattctggacgctaacatcgtgcaggctcagctccagctcgaactcaaagaatccctgcgatggtggcgaaacacctgcttcgtcgagaagctgcctttcgcgcgagaccgactgattgagtcttacttctggtctaccggtatggtcgagccccgacagcacgctaacgcccgaattattatggctaaggtcatcgccctgatcaccgtcatggacgacatctatgacgtgtacggaaccttagaagagctggagcagttcaccgaggctttccgacgatgggacgtgtcttctattgaccagctccctacctacatgcagctgtgcttcctggctattaacaacttcgtggacgacaccgcttacaacgtgctgaaagaatccggcgtcaacgtgatgacctacctgcgaaagtcttgggtggaccaggctgagaactacctcatggagtctaagtggtactactccggccacaagccttctctggaggaatacctcgaaaactcttggatttctgtgtccggtccttgcgtcctcacccacgagttcttcggcgtcaccgactctctcgctaaggacaccctggactctctgtacgagtaccacgacattgtgcgatggtcctcttacctgctgcgactggctgacgacctcggaacctccgtcgaggaagtgtcccgaggtgacgtgcctaagtctattcagtgctacatgcacgacaacgacgcttctgaagaggaagcccgacagcacattaagggcctcatccgagagatgtggaagaagatgaacgtcgagcgagtgtctgaggactcccctttctgccgagacttcattcgatgctgcgaggacctcgggcgaatggctcagttcatgtaccactacggtgacggtcacggaacccagcaccccaagatccaccagcagattgccgcttgcctgttccagcctttcgccTAG

Truncated *NDPS1* gene

ATGtccgcccgaggtctcaacaaaatctcttgctccctcaacctacagaccgagaagctgtgctacgaggacaacgacaacgacctcgacgaagagctgatgcctaagcacatcgctctcatcatggacggaaaccgacgatgggccaaggacaagggtctggaggtgtacgagggccacaagcacattattcctaagctgaaggaaatttgcgacatttcttctaagctggggatacagattattaccgctttcgctttctctaccgagaactggaagcgatctaaggaagaggtggacttcctgctccagatgttcgaggaaatctacgacgagttctctcgatccggcgtgcgagtgtccattatcggatgcaagtctgacctccccatgaccctccagaagtgcatcgccctcaccgaagaaaccaccaagggtaacaagggtctgcacctcgtgattgctctgaactacggcggatactacgacattctccaagctaccaagtccatcgtcaacaaggctatgaacggactgctggacgtggaggacattaacaagaacctgttcgaccaagaattggaatctaagtgccccaaccctgacctgctcatccgaaccggcggtgagcagcgagtgtctaacttcctcctgtggcagctggcttacaccgagttctacttcaccaacaccctgttccctgacttcggtgaagaggatctgaaggaagctatcatgaacttccagcagcgacaccgacgattcggtggccacacctacTAG
